# Mitochondrial Dysfunction: Pathophysiology and Mitochondria-Targeted Drug Delivery Approaches

**DOI:** 10.3390/pharmaceutics14122657

**Published:** 2022-11-30

**Authors:** Tanzeel Khan, Rashid Waseem, Zainy Zehra, Ayesha Aiman, Priyanka Bhardwaj, Jaoud Ansari, Md. Imtaiyaz Hassan, Asimul Islam

**Affiliations:** 1 Centre for Interdisciplinary Research in Basic Sciences, Jamia Millia Islamia, New Delhi 110025, India; 2 Department of Biosciences, Jamia Millia Islamia, New Delhi 110025, India

**Keywords:** mitochondrial dysfunction, nanoparticles, drug delivery, pathophysiology

## Abstract

Mitochondria are implicated in a wide range of functions apart from ATP generation, and, therefore, constitute one of the most important organelles of cell. Since healthy mitochondria are essential for proper cellular functioning and survival, mitochondrial dysfunction may lead to various pathologies. Mitochondria are considered a novel and promising therapeutic target for the diagnosis, treatment, and prevention of various human diseases including metabolic disorders, cancer, and neurodegenerative diseases. For mitochondria-targeted therapy, there is a need to develop an effective drug delivery approach, owing to the mitochondrial special bilayer structure through which therapeutic molecules undergo multiple difficulties in reaching the core. In recent years, various nanoformulations have been designed such as polymeric nanoparticles, liposomes, inorganic nanoparticles conjugate with mitochondriotropic moieties such as mitochondria-penetrating peptides (MPPs), triphenylphosphonium (TPP), dequalinium (DQA), and mitochondrial protein import machinery for overcoming barriers involved in targeting mitochondria. The current approaches used for mitochondria-targeted drug delivery have provided promising ways to overcome the challenges associated with targeted-drug delivery. Herein, we review the research from past years to the current scenario that has identified mitochondrial dysfunction as a major contributor to the pathophysiology of various diseases. Furthermore, we discuss the recent advancements in mitochondria-targeted drug delivery strategies for the pathologies associated with mitochondrial dysfunction.

## 1. Introduction

Mitochondria are among the most important organelles in eukaryotic cells and have a distinctive structure composed of lipid-bilayer membranes [1]. A mitochondrion has a unique structure comprising four parts: the outer mitochondrial membrane (OMM), the inter-membranous space (IMS), the inner mitochondrial membrane (IMM), and the matrix, with each part performing a specific role. The permeability of mitochondrial lipid membranes differs; the outer membrane is permeable to a broad range of small molecules, but the inner membrane is selective [2]. The passage of molecules through the IMM is controlled by a variety of specialized channel proteins [3]. Therefore, compared to the cytoplasm, the mitochondrial matrix has a remarkably different electrochemical potential and composition. Moreover, mitochondria are the only organelles that have their own genomes, i.e., a circular form of DNA with 16,500 circular base pairs and 37 genes. These mitochondrial DNAs (mtDNAs) encode 2 ribosomal RNAs (rRNAs), 13 messenger RNAs (mRNAs), and 22 transfer RNAs (tRNAs), which are all required for the synthesis of 13 proteins that are components of the electron transport chain (ETC) for performing oxidative phosphorylation [4]. Mutations in either mtDNA or nuclear DNA genes coding for mitochondrial proteins may lead to the onset of mitochondrial diseases [5,6]. The identification of mitochondria as an emerging pharmaceutical target has led to the development of several mitochondria-targeting strategies for the effective treatment of diseases associated with mitochondrial dysfunction. Some of the current drugs’ limitations include low solubility, non-selective biodistribution, and poor bioavailability. Nanopreparations have the potential to overcome the present barriers by providing a sustained and targeted medication delivery system to mitochondria. Recently, NPs and traditional chemotherapeutic drugs have been conjugated to create biocompatible, multifunctional mitochondria-targeted nanoplatforms. Furthermore, nanopreparations may also improve therapeutic compounds’ pharmacokinetic characteristics and bio-distribution patterns. This technique is also being utilized to create targeted medicine delivery systems and hybrid nanostructures that can be activated by light (also known as photodynamic and/or photothermal therapy). In this review, we have discussed the role of mitochondrial dysfunction in the pathophysiology of various diseases. Further, we have also focused on mitochondria-based therapy involving different targeting mechanisms and the current approaches for mitochondria-targeted drug delivery.

## 2. Physiological Importance of Mitochondria

### 2.1. Mitochondria and Oxidative Phosphorylation

Mitochondria are implicated in various critical processes in animal cells, such as oxidative phosphorylation (OXPHOS), the tricarboxylic acid (TCA) cycle, fatty-acid oxidation, calcium ion homeostasis in association with the endoplasmic reticulum (ER) [7], amino acid metabolism [8], and the regulation of apoptosis [9]. The production of ATP for energy is the primary function of mitochondria. There are two ways by which cells produce ATP; in the cytosol through glycolysis and in mitochondria by oxidative phosphorylation. Substrates such as pyruvate and fatty acids are oxidized through TCA and β-oxidation pathways, respectively. The by-products of both the processes, flavin adenine dinucleotide (FADH_2_) and nicotinamide adenine dinucleotide (NADH) are used by the electron transport chain (ETC) of mitochondria to generate ATP. The ETC comprises protein complexes that lie within the inner mitochondrial membrane [10]. The electrons transported by NADH and FADH2 are transferred to complex I (NADH dehydrogenase) and complex II (succinate dehydrogenase) of protein complexes. After that, these electrons are transported by coenzyme Q to complex III (cytochrome bc1) and finally through complex IV (cytochrome c oxidase) to oxygen molecules. This sequential passage of electrons along these protein complexes is accompanied by the generation of a proton gradient across the IMM, which is further utilized by F_O_ F_1_ ATP synthase in the formation of ATP. Therefore, it is clear that any damage that impairs the mitochondrial capacity to carry out these critical functions will have a significant impact on ATP synthesis that would be detrimental to cellular functioning [11].

### 2.2. Mitochondria and Reactive Oxygen Species (ROS)

ROS are produced as a consequence of oxygen metabolism, which includes hydrogen peroxide (H_2_O_2_), superoxide anions (O_2_^−^), and hydroxyl radicals (OH^●^) [12]. These ROS are mainly generated from oxidative phosphorylation. The primary member of ROS is a superoxide anion (O_2_^−^) produced by both complex I and complex III of the ETC [13]. These overproduced superoxide anions are endogenously controlled by superoxide dismutase (SOD) via their conversion into hydrogen peroxide, which in turn is converted into water by catalase or peroxidase enzymes. However, in various diseases such as neurological diseases, cardiovascular disorders, and autoimmune diseases, a disturbance of this redox balance occurs in mitochondria, which activates inflammasomes, RIG-I-like receptors (RLRs), and mitogen-activated protein kinases (MAPK), leading to the activation of innate immune and inflammatory responses [14]. Numerous anti-oxidants such as coenzyme Q10 [15], vitamin E [16], apocynin [17], and SOD mimetic [18] in conjugation with small cationic molecules such as triphenylphosphonium (TPP^+^) have been used in controlling imbalanced redox species in mitochondria.

### 2.3. Mitochondria and Calcium Homeostasis

The endoplasmic reticulum in cells is primarily responsible for storing calcium cations; however, mitochondria can also temporarily store calcium [19]. In different kinds of healthy cells, mitochondria can withstand intracellular calcium concentrations between 50 and 500 nM. This buffering capacity is maintained via the calcium uniporter located in the IMM [20]. Ca^2+^ ions can permeate through the outer mitochondrial membrane; when the Ca^2+^ ion concentration surpasses a 1 mM concentration in extreme conditions, the calcium uniporter channel opens and transfers Ca^2+^ ions from the cytosol to the matrix of mitochondria [21]. Calcium homeostasis is important for various metabolic functions. Calcium is intricately involved in synaptic plasticity, organelle movement, and neurotransmitter vesicle release in brain [22]. In cellular signaling pathways, Ca^2+^ ions are critically involved and balance cellular signaling among cells [23].

### 2.4. Mitochondria and Apoptosis

A highly controlled type of cell death called apoptosis is managed by mitochondria. It is a crucial process in the development (e.g., in the modeling of limbs and neurodevelopment) and lifelong maintenance of tissue homeostasis. In terms of morphology, cells undergoing apoptosis show membrane blebbing and chromatin condensation. Therefore, apoptosis can also be easily characterized. The mitochondrial pathway (extrinsic) and the death receptor pathway are the two pathways through which apoptosis manifests itself in mammalian cells [24]. The mitochondrial apoptosis pathway can respond to both intracellular and extracellular cues, as exemplified by DNA damage. Cytochrome c, which typically transports electrons between complexes III and IV of the ETC, is the most effective signaling molecule in the apoptotic pathway. In apoptosis, however, cytochrome c release leads to the loss of mitochondrial membrane potential, resulting in the permeabilization of the OMM. This release of cytochrome *c* from the mitochondrial intermembrane space to the cytosol activates various caspase enzymes that cause apoptosis [25].

### 2.5. Mitochondria and Fe/S Clusters

The biosynthesis of various protein cofactors, including Moco, heme, lipoic acid, biotin, and iron–sulfur (Fe/S) clusters, is another important function of mitochondria [26]. Among these, Fe/S clusters are of particular importance due to their involvement in electron transfer reactions as well as in catalytic and regulatory processes. Moreover, they also serve as sulfur donors during the synthesis of lipoic acid and biotin. There are many types of Fe/S clusters, but [2Fe-2S] and [4Fe-4S] are the most prevalent and simplest clusters [27]. Mitochondrial Fe–S biosynthesis is initiated by the iron–sulfur cluster (ISC) assembly, which consists of more than 15 components [28]. Apart from mitochondrial Fe/S, this iron–sulfur cluster (ISC) assembly machinery in mitochondria is also required for the biosynthesis of cytosolic Fe/S clusters [29]. In mitochondria, these Fe/S proteins are specifically involved in the TCA cycle (aconitase), fatty acid oxidation (ETF-ubiquinone oxidoreductase), the electron transfer chain (respiratory complexes I–III), and in biotin and lipoate biosynthesis (lipoate and biotin synthases) [29]. Dysfunction in assembly with respect to the formation of Fe/S proteins is linked with severe and frequently fatal neurodegenerative, metabolic, or hematological diseases [30,31].

## 3. Role of Mitochondrial Dysfunction in Pathophysiology 

The functioning of every enucleated cell in the human body depends on healthy mitochondria. A disturbance of the basic mitochondrial functions such as bioenergetic functions, the regulation of oxidative stress, and the homeostasis of ions is typically characterized as mitochondrial dysfunction. In the process of an ETC, the release of ROS from the mitochondria causes many detrimental effects, such as mtDNA/RNA damage, lipid oxidation, protein oxidation, the activation of the Ca^2+^-dependent mitochondrial permeability transition pore, and the release of cytochrome c, which causes the formation of apoptosomes and eventually leads to apoptosis. Both the oxidative stress-related and hereditary mitochondrial DNA abnormalities result in mitochondrial dysfunction, which triggers the cascade of cell death signals leading to organ failure and diseases. Numerous diseases, including diabetes [32], heart failure and ischemia-reperfusion damage [33], cancers [34], Alzheimer’s, and Parkinson’s disease [35], have recently been linked to mitochondrial dysfunction. Therefore, numerous studies are being undertaken to clarify the way in which mitochondrial dysfunction relates to the pathophysiology of different diseases. Herein, we have discussed the pathophysiology of mitochondrial dysfunction in various diseases.

### 3.1. Mitochondrial Dysfunction and Cancer

Several past investigations have demonstrated that mitochondria play a key role in the etiology of several ailments, including cancer. There are several reports suggesting the involvement of mitochondrial dysfunction in tumors or cancers [34,36]. The commonly found pathologies include reduced oxidative phosphorylation/ATP synthesis, reactive oxygen species over-production, altered calcium homeostasis, and a surge in inflammation [37]. When compared to normal cells/tissues, cancer cells are more prone to fluctuations in energy metabolism. Faster cell proliferation, longevity, and the reoccurrence of tumor cells are also linked to mitochondrial dysfunction. Furthermore, it has been repeatedly observed that mitochondrial failure results in physiological conditions such as hypoxia and acidosis in cancer patients [38]. In addition, there are studies reporting that cancer cells usually have a higher membrane potential of 220 mV than the 140 mVof normal cells [39,40], which may lead to enhanced anabolism, irresponsiveness towards anti-growth factors, and, most importantly, unregulated apoptosis and futile autophagy [41]. Several notable differences in the structure and function of mitochondria between normal and cancer cells have been revealed. A few of the key differences are listed below.

#### 3.1.1. Metabolic Alterations Associated with Cancer

In cancer, various alterations in the functions of mitochondria have been reported, viz., lactic acid over-production accompanied by decreased pyruvate oxidation [42], enhanced glutaminolytic activity [43], gluconeogenesis increment [44], and lower fatty acid oxidation [45]. The bioenergetics guided by mitochondrial functions show a metabolic anomaly in the case of tumor cells in terms of respiratory substrate selection, calcium-buffering capability, or the reduced catalytic activity of mitochondrial enzymes (e.g., Cytochrome c oxidase) [46].

#### 3.1.2. Structural Differences Associated with Cancer

The lipid distribution of inner membrane of a tumorous cell has a higher percentage of cholesterol, altered total phospholipid content, and/or variations in the number of individual phospholipids [47]. When compared to their non-malignant counterparts, cancer cells display variations in the structure and/or relative distribution of numerous proteins. A typical example includes the altered function of F1 ATPase in hepatic cells due to a significant decline in the levels of the b subunit of the F1 component of mitochondrial ATPase accompanied by the up-regulation of ATPase inhibitor protein (IF 1) [48,49]. Conversely, the expression of an pro-apoptotic IMM protein called BAX is decreased in some cancer cell lines [50,51].

#### 3.1.3. OXPHOS Pathway Differences Associated with Cancer

OXPHOS is the process by which ATP is generated in normal cells. However, certain cancer-causing agents, such as radiation, carcinogens, and/or oncogenes, convert normal cells to cancer cells, and these cancer cells subsequently alter the ATP generation process from OXPHOS to Glycolysis [52]. This switch in the metabolic pathway is associated with variations in the normal values of several elements such as mitochondrial membrane potential (MMP; m), glutathione (GSH), pH, and reactive oxygen species (ROS) [53,54].

#### 3.1.4. Physiological Differences Associated with Cancer

In normal cells, ATP production consumes the majority of mitochondrial oxygen, only leaving a small percentage for other processes such as ROS production, whereas cancer cells have significantly higher mitochondrial oxygen levels than normal cells due to the hypoxic extra-tumoral environment [53]. This is owning to the fact that there is reduced ATP production in the mitochondria of tumor cells, which, in turn, results in a 2.5-fold lower oxygen consumption rate (OCR) than that of the mitochondria of healthy cells [53]. 

#### 3.1.5. Necrosis Associated with Cancer

The role of mitochondria in necrotic cell death has long been recognized [55]. A few situations, most notably oxidative stress and calcium build-up inside mitochondria, can cause a high-conductance leak resulting in the opening of IMM [56]. As a consequence, the electrochemical proton gradient falls, halting ATP synthesis followed by activating ROS generation. In addition, some physiological conditions of necrotic cell death like pH-dependent ischemia/reperfusion injury have also been reported to cause mitochondrial failure [57,58]. Tumor cells were able to strategically prevent hypoxia-mediated cell death by down-regulating p53, a cancer suppressor protein that controls cellular stress response [59]. 

### 3.2. Mitochondrial Dysfunction and Neurodegenerative Diseases

**Figure 1:**
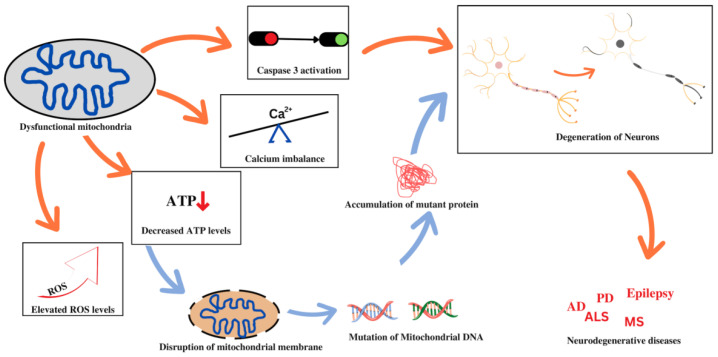
Mitochondrial dysfunction and associated neurodegenerative disorders.

The aberrant folding and consequent accumulation of proteins within the cell body of neurons are indications of neurodegenerative disorders. This is plausible due to perturbations in mitochondrial function that can have profound repercussions on the structure and functioning of the neurons, thereby resulting in neurodegeneration. Some of these disorders will be discussed in this section in relation to mitochondrial dysfunction. A vast number of studies indicate that impaired brain metabolism or mitochondrial dysfunctions are some of the best-documented anomalies and early signs in brains affected by major neurodegenerative disorders. Mitochondrial dysfunction associated with non-maternal inheritance has been extensively reported in several neurological diseases, most notably amyotrophic lateral sclerosis (ALS) [60], Parkinson’s disease (PD) [61], Alzheimer’s disease (AD) [62], multiple sclerosis [63], schizophrenia [64], epilepsy [65], neuropathic pain, and Huntington’s disease (HD) [65]. Figure 1 depicts the association between mitochondrial dysfunction and various neurodegenerative diseases. Despite the varying mode of transmission and the unknown specific cause, the common and remarkable signature of mitochondrial dysfunction in such diseases is the malfunctioning of the respiratory chain, resulting in a variety of clinical manifestations. Other causes of mitochondrial malfunction and abnormal mitochondrial morphology include mtDNA mutations [66], oxidative damage, and mitochondrial protein aggregation [67]. The imbalance between ROS production and oxidation leads to oxidative stress, which disrupts the functioning of mitochondrial respiratory chain, affects calcium homeostasis, alters membrane permeability, increases heteroplasmic mt DNA levels, and weakens mitochondrial defense systems [68,69]. Oxidative stress may damage cellular components of mitochondria such as proteins, nucleic acids, and lipids, and contribute to the production of intracellular ROS, leading to mtDNA mutations in neurodegenerative disorders. These involve a variety of proteins that regulate oxidative phosphorylation (OXPHOS) and mitochondrial dynamics and are thus involved in regulating the integrity of the mitochondrial structure. Furthermore, mitochondrial failure largely affects mitochondrial biogenesis and dynamics, which are both associated with a variety of age-related neurodegenerative disorders [70]. In this section, we have briefly explored the role of mitochondrial dysfunction in various neurodegenerative diseases.

#### 3.2.1. Alzheimer’s Disease

Alzheimer’s disease is the most common cause of dementia and is becoming one of the most costly, fatal, and burdensome diseases of the 21st century [71,72,73]. Its pathological hallmarks include nerve cell degeneration, the appearance of neurotic plaques, and neurofibrillary tangles [74,75,76]. Even though the precise mechanism of AD pathogenesis remains unknown, increasing evidence implies that mitochondrial dysfunction and oxidative stress play a significant role in the disease’s etiology [62]. Previous research has found that mtDNA and enzyme abnormalities in the brains of AD patients were followed by the altered morphology and mass of the organelle [77]. These patients were found to have altered mitochondrial enzymatic activity in their brains that led to impaired mechanisms of OXPHOS and the tricarboxylic acid (TCA) cycle [78,79], which further results in low ATP production and an increase in oxidative stress [80,81]. According to the most widely accepted explanation, tau (τ) and Aβ (amyloid β) damage neuronal cells in AD by interfering with the supply of energy and the antioxidative response, resulting in mitochondrial and synaptic dysfunction. The α-ketoglutarate dehydrogenase enzyme complex (α-KDHC) is a crucial mitochondrial enzyme for oxidative metabolism, and its activity diminishes with age. The mechanism of its inactivation is unknown; however, Q. Shi et al. have found that α-KDHC function can be restored, and can be a viable therapeutic method for Alzheimer’s disease [82]. Another study indicates that AD disrupts mitochondrial biogenesis as well as the dynamics of fission and fusion, resulting in an unequal distribution of mitochondria in the neurons [83]. Aβ is thought to be responsible for activating downstream cascades in microglia that cause mitochondrial dysfunction and exacerbate inflammation and cytotoxicity in AD patients [84]. Thus, investigations explaining the operative pathways of mitochondrial anomalies in AD may aid in a better understanding of the etiology of this neurodegenerative illness and may assist in the advancement of therapeutic options to safeguard synaptic activity and consequent cognitive function.

#### 3.2.2. Parkinson’s Disease

The role of mitochondrial dysfunction as an activator, propagator, or bystander in Parkinson’s disease (PD) has been a mystery for decades. PD is the second most prevalent neurodegenerative disorder in the world, and it, like AD, has both hereditary and environmental risk factors [85]. The continuous loss of dopaminergic neurons as well as the aggregation of fibrous protein such as α-synuclein deposits in the cytoplasm of neurons (i.e., Lewy bodies) and nerve fibers (i.e., Lewy neurites) in the substantia nigra of the brain are pathological signatures of PD [61]. This disease manifests both motor and non-motor symptoms, wherein some motor symptoms include myotonia, rest tremors, hypokinesia, and aberrant posture [86], while non-motor symptoms include anxiety, sadness, constipation, frequent micturition, sleep behavior disorder (SBD), complications in rapid eye movement (REM), and cognitive difficulties [87]. In rare cases, PD is caused by mutations in the PINK1 (PTEN-induced kinase 1) or PRKN (parkin RBR E3 ubiquitin protein ligase) genes, which affect the selective autophagic clearance of damaged mitochondria (mitophagy) [88]. According to F. Wauters et al., the most prevalent monogenic cause of PD is a mutation in the gene encoding LRRK2 (leucine-rich repeat kinase 2) [89]. RAB10, a GTPase-activating protein (GAP) and a substrate of LRRK2, accumulates on the depolarized mitochondria via signals from activated PINK1 and PRKN genes. It further binds the autophagy receptor OPTN (optineurin), which enhances OPTN accumulation on depolarized mitochondria and aids mitophagy [90]. Moreover, several studies have also suggested that mtDNA mutations [91,92] and abnormalities in complex I of the mitochondrial ETC [93] play an essential role in the etiopathogenesis of PD. According to theories put forth by researchers [94], the substantia nigra—a group of dopaminergic neurons of the midbrain that undergoes age-related neurodegeneration in PD patients, has high oxidative capacities, and that seems to be particularly sensitive to mitochondrial dysfunction [95], loss in the respiratory chain and, subsequently, high oxidative stress—that is uniquely susceptible to the accumulation of somatic mtDNA mutations and deletions over time [96]. Post-mortem samples revealed that the levels of somatic mtDNA deletions in the substantia nigra of PD patients were marginally higher than those in controls of a comparable age [96] and other neuropathological disorders [97]. Consequently, deletions may result from a compromised mitochondrial replication system, specifically, from mitochondrial DNA polymerase γ (POLG) mutations in the mitochondrial polymerase [98]. In accordance with these findings, it is essential to explore drug development strategies to prevent mtDNA dysfunction as a potential way in which to slow the progression of PD.

#### 3.2.3. Multiple Sclerosis

Multiple sclerosis (MS) is a metabolically dependent neurodegenerative disorder that is caused by persistent axonal loss of the brain and the spinal cord, which thereby initiates cognitive decline and physical disability among the affected. This chronic and progressive autoimmune disease has a complex pathophysiology that is characterized by flare-ups of inflammation and the breakdown of the myelin sheath (an insulating layer of fat that protects nerve fibers) in the central nervous system (CNS) [99]. A disrupted myelin sheath may result in the blocking or impedance of nerve signals and hamper the control of various physiological functions such as vision, sensation, muscle coordination, and strength. In the past several years, it has become obvious that malfunctioning mitochondria are key contributors to axonal and neuronal damage [100]. Animal and histological investigations indicate that invading leukocytes and activated microglia play a critical role in neuronal mitochondrial dysfunction [63]. Disruptions in mitochondrial function were accompanied by significant alterations in the morphology and density of the organelle, which cause inflammatory lesions in MS patients. However, at the onset of disease manifestation, the biochemical activity of mitochondrial complex I was specifically compromised in these spinal tissues [101]. So, it can be concluded that these mechanisms create an imbalance in energy and further contribute to irreversible impairment and neurodegeneration. Thus, extensive research and multiple mitochondria-targeted neuroprotective treatments must be developed as a part of the standard MS treatment regimen.

#### 3.2.4. Amyotrophic Lateral Sclerosis 

Amyotrophic lateral sclerosis (ALS) is a motor neuron disease (MND) characterized by the loss of upper motor neurons in the motor cortex and lower motor neurons in the brain stem and the spinal cord. Progressive limb degeneration results in muscular atrophy, paralysis, and, eventually, the death (respiratory failure) of the patient [102]. From a genetic standpoint, ALS can be of two types, namely, familial and sporadic, the latter one being most common (~90%) amongst affected individuals [103]. Further, the pathology of the aforementioned disease includes mitochondrial degeneration, oxidative stress, glutamate excitotoxicity, decreased axonal transport, glial cell disease, and defective RNA metabolism [102]. Thiol is oxidized during mitochondrial dysfunction, resulting in a cascade of multiple processes such as calcium imbalance and the breakdown of the mitochondrial membrane promoting cell death [104]. Analysis of ALS models revealed that the build-up of aberrant mitochondria in motor neuron axons leads to defective mitochondrial transport in ALS [104]. Furthermore, mitochondrial damage limits NAD^+^ and ATP production while increasing ROS production, leading to mtDNA mutations, structural deformity, aberrant protein aggregation, and, eventually, the loss of motor neurons [104]. Additionally, cell culture and animal investigations have revealed faulty calcium homeostasis and ROS overproduction in relation to abnormal oxidative metabolism in mitochondria [105]. Moreover, abnormalities in mitochondrial dynamics and the interruption of axonal transport were also identified in ALS models [106]. As a result, mitochondrial degradation is determined to be the cause of most familial or sporadic ALS cases and addressing ALS-associated mitochondrial deregulation pharmacologically may present a possibility for delaying disease progression.

### 3.3. Mitochondrial Dysfunction and Cardiovascular Diseases (CVD)

In 2019, Asia accounted for 58% of the 18.6 million CVD deaths globally [107]. Many cardiac illnesses, including atherosclerosis, ischemia-reperfusion injury, heart failure, and hypertension, are thought to be accompanied by mitochondrial dysfunction, most likely as a result of insufficient cellular energy production and unchecked ROS production. mtDNA damage is a significant contributor to mitochondrial dysfunction and is a key phenotypic feature [6]. In these cases, apoptosis, inflammation, fibrosis, and cardiac remodeling are stimulated, and sarcomere protein function is impaired. Other characteristics of mitochondrial dysfunction include decreased numbers of mitochondria in tissues, the stimulation of apoptosis and inflammation, the absence or dysfunction of mitochondrial enzymes, and impaired mitochondrial biogenesis [108,109]. In this section, we have briefly discussed the role of mitochondrial dysfunction in various cardiovascular diseases.

#### 3.3.1. Atherosclerosis

Atherosclerosis, a chronic inflammatory disease, is characterized by the accumulation of lipids, primarily cholesterol, and other compounds such as fatty materials, cellular waste products, calcium, and fibrin within the artery wall [110]. The progression of atherosclerosis depends on numerous factors and one of its hallmarks is ROS overproduction, which is involved in various processes. Proteins, lipids, and nucleic acids can suffer oxidative damage from prolonged exposure to ROS or excessive ROS production [111]. Endothelial nitric oxide synthase (eNOS) converts L-arginine into L-citrulline to produce the majority of the nitric oxide (NO) produced in the endothelium. Intracellular arginine levels, which are, in turn, governed by mitochondrial arginase II, are the primary regulators of NO levels. As a result, NO needs a functioning mitochondrial respiratory chain (MRC) to maintain proper levels [112]. The major causes of decreased NO concentrations in the cell are eNOS breakdown brought on by elevated ROS levels and the loss of mitochondrial membrane potential. Lower NO secretion and production are the initial signs of endothelial change in early atherosclerosis [113]. Improper mitophagy also increases mitochondrial damage and the release of ROS, mtDNA, and K+ into the cytoplasm, which encourages NLRP3 inflammasome activation. Mitophagy is a biological mechanism involved in mitochondrial rejuvenation. In fact, the NRLP3 protein can directly interact with released mtDNA to start an inflammatory response [114]. 

#### 3.3.2. Ischemic Stroke

Strokes continue to number among the major causes of death in developed nations and constitute the primary cause of physical and intellectual disability in adults. Stroke is still the most common cause of mortality in affluent countries and the main factor in adult physical and intellectual disability [115,116]. When the blood supply to the brain tissue provided by blocked arteries is reduced, an ischemia event takes place. Cell death ultimately results from altered cellular homeostasis caused by a scarcity of oxygen and nutrients [117,118,119]. Various enzymes have been shown to play a role in ischemia [120]. Numerous isoforms of nitric oxide synthase (NOS) activated post-ischemic stroke, such as neuronal, endothelial, and inducible NOSs, mediate excessive NO production [121,122,123,124]. The B-cell lymphoma (BCL-2) protein family is a significant regulator of the permeability of the OMM and is essential for the intrinsic apoptotic pathway [125]. BCL-2 may have a significant role in the regulation of neuronal death in cerebral ischemic strokes according to several reports [126,127,128,129,130]. 

Aside from the BCL-2 pathway, several other major apoptotic pathways involve the release of pro-apoptotic factors such as apoptosis-inducing factor (AIF) and the second mitochondrion-derived activator of caspase (SMAC) from the mitochondria. The pro-apoptotic factor SMAC releases from mitochondria and binds to the X-chromosome-linked inhibitor of apoptosis protein (XIAP), suppressing its anti-apoptotic activity and preventing serial procaspase activation and thus further inducing apoptosis following cerebral ischemia [131,132]. Another major pro-apoptotic factor, AIF, is a mitochondrial protein which was discovered to be a caspase-independent modulator of the degradation phase of apoptosis. AIF was proposed to serve as a mitochondrial effector of apoptotic cell death after its transfer from the mitochondria to the nucleus [133]. BH3-interacting domain death agonist (Bid) is a cytosolic protein that induces the release of cytochrome c, which leads to programmed cell death. The Bid protein has been demonstrated to maintain AIF in the nuclei, which speeds up and strengthens the apoptotic process [134]. Moreover, AIF has also been proven to block poly (ADP-ribose) polymerase. Experiments performed on animal models of ischemic stroke show the translocation of AIF with apoptotic DNA fragmentation, which happens prior to or concurrently with the release of cytochrome c from mitochondria [135]. Furthermore, the role of AIF is also seen to govern neuronal death brought about by oxygen-glucose deprivation, glutamate-induced toxicity, and experimental ischemic stroke in vivo [134].

## 4. Strategies for Mitochondria-Targeted Therapy

**Figure 2:**
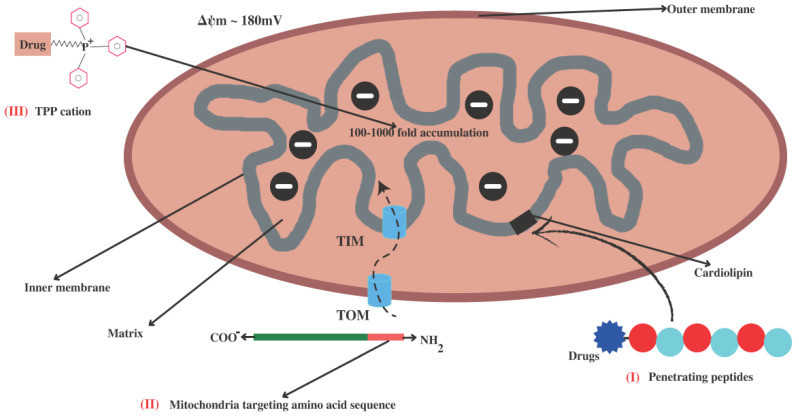
Different strategies for mitochondria-targeted drug delivery. (I) Peptides conjugated with drugs imported to the mitochondria through binding with phospholipid cardiolipin (CL). (II) Cellular proteins with N-terminal mitochondrial targeting sequences are imported into mitochondria via TOM and TIM channels. (III) Mitochondrial uptake of drug-conjugated lipophilic cations such as triphenylphosphonium (TPP^+^) occurs due to difference in transmembrane potential (ΔΨm) of mitochondria.

The identification of mitochondria as an emerging pharmaceutical target has led to the development of several mitochondrial targeting strategies for the effective treatment of diseases based on mitochondrial dysfunction. The primary challenge in exploiting mitochondria as a target is the delivery of therapeutic molecules. As we described above, permeability of both the membranes in mitochondria is different. The transition pore in the OMM is wider, and it is through this pore that therapeutic molecules can easily traverse. Whereas the highly folded hard IMM has narrower transition slits that separate the intermembrane space and the mitochondrial matrix, making it difficult for many therapeutic molecules to cross the mitochondrial matrix. Thus, research has been directed towards improving therapeutic delivery and reducing the unwanted effects of the delivered drugs. Numerous drug delivery approaches are being developed in light of the specific properties of mitochondria, including their membrane potential and lipophilicity [136], specialized protein import machinery [137], and distinctive phospholipid composition in the IMM [138], as shown in Figure 2. The first characteristic of a mitochondrion is that it contains a negative charge. The potential (ΔΨm) between the matrix and the intermembranous space is around 180 mV; mitochondria exploit this potential as a proton-motive force for ATP production [139]. However, this ΔΨm was exploited for mitochondrial targeting by using positively charged ions (cations) that are attracted to the mitochondria via electrostatic forces. The second distinguishing feature of mitochondria is the unique structure of their IMM and lipid composition, which mostly consists of cardiolipin and may be exploited for targeted drug delivery to the mitochondria. The phospholipid cardiolipin plays a key role in apoptosis and provides structural support for respiratory chain complexes [140].

Mitochondria generally lose most of their genetic material while forming endosymbiotic interactions with the host eukaryotic cell, forcing them to require proteins encoded in the nuclear genome. As a consequence, mitochondria possess protein import machinery that identifies proteins with a specific amino acid sequence [141]. Thus, drug delivery to mammalian mitochondria is accomplished using one of these approaches, or by a combination of both. Herein, we have discussed various molecules that are being used for drug delivery in mitochondria.

### 4.1. Small Lipophilic Cationic Molecules Targeting Mitochondria 

#### 4.1.1. Triphenylphosphonium Cation (TPP^+^)

**Figure 3:**
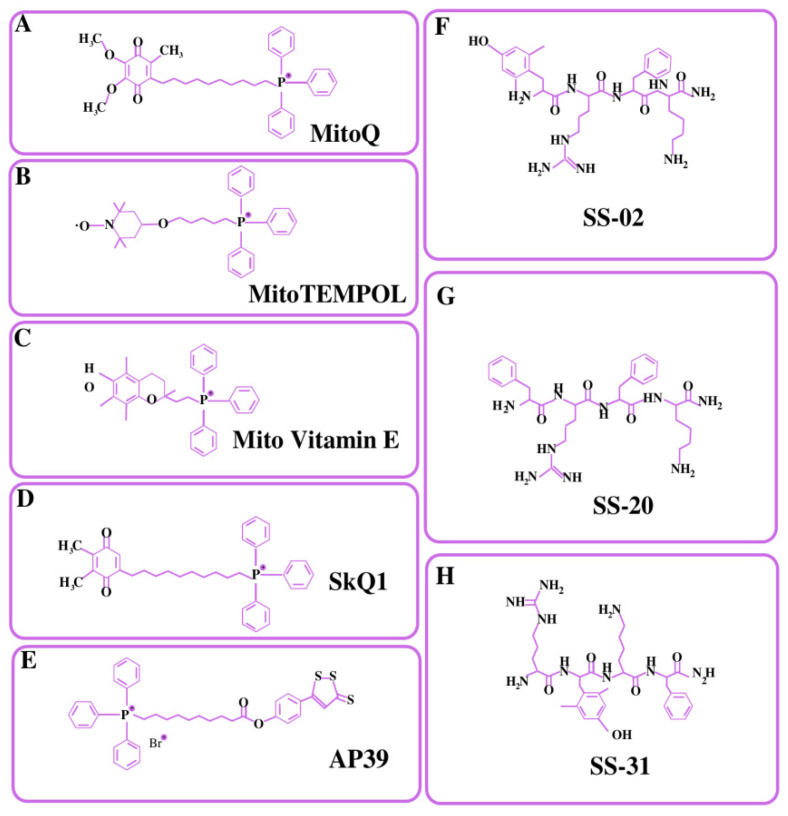
Structural formulas of major types of mitochondria-targeted compounds. (**A**–**E**) TPP^+^-conjugated mitochondrial targeted compounds. (**F**–**H**) Membrane-penetrating peptides.

In mitochondria, there is a significant transmembrane potential of 140–180 mV (negative inside) that may be used to transport positively charged molecules to mitochondria [142]. Murphy and colleagues pioneered the use of lipophilic cations as mitochondrial carriers, which are also known as mitochondriotropic ligands [143]. TPP^+^ is a lipophilic cation, and the potential gradient created by its charge dispersion over the surface area causes its accumulation inside the mitochondrial matrix [144]. The percentage of the TPP^+^ concentration inside the negatively charged membrane compartments rises by one order of magnitude for every 60 mV of negative membrane potential. The plasma membrane voltage typically varies between −30 and −60 mV, which is enough to cause an up to 10-fold buildup of TPP within the cell. Typical mitochondria, on the other hand, have a membrane potential of −180 mV, which facilitates an increased accumulation of TPP^+^ inside the mitochondria by 1000 times [144]. TPP^+^ aids in the delivery of mitochondrial drugs such as AP39 [145], Mito-Vit-E [136], Mito-Q [146], SkQ1 [147], and 2,2,6,6-tetramethyl-4-[5-(triphenylphosphonium) pentoxy] piperidine-1-oxybromide (Mito-TEMPOL) [148]. The structural formulas of all these drugs have been depicted in Figure 3. AP39 is mitochondria-targeting motif that comprises TPP+ attached to a H_2_S donor moiety (dithiol-77 ethione) through an aliphatic linker. There is emerging evidence regarding the roles of H_2_S in reducing the release of mitochondrial death signals and maintaining mitochondrial integrity [149]. Several studies suggested that AP39 are capable of preventing hyperglycemia-induced oxidative stress [150], reducing amyloid-β deposition in the brain, and ameliorating spatial memory deficits in APP/PS1 mice [151]. Mito-Vit-E was made by combining vitamin E with TPP^+^, with the latter driving the compound in mitochondria. Mito-Vit-E is an anti-oxidant that has been shown to revive mitochondrial activity and prevent cell death [136]. Mito-Q is produced through the conjugation of ubiquinone with TPP^+^ and is commonly utilized as an antioxidant to prevent cell death. SkQ1 are the derivatives of plastoquinone attached via the C10 hydrophobic linker to the TPP cation. SkQ1 has shown greater anti-oxidant activity and binding affinity with cardiolipin as compared to mito-Q, and has shown decreased protein oxidation, the prevention of cell apoptosis, decreased ROS levels, and the prevention of lipid peroxidation [147]. Several studies reported that SkQ1 showed high efficacy in a wide variety of eye diseases such as glaucoma, retinopathy, and dry eye syndrome [152,153]. Mito-TEMPOL is a well-known superoxide scavenger that targets mitochondria. Mito-TEMPOL therapy may reduce ATP depletion-induced necrosis and apoptosis by maintaining mitochondrial integrity and lowering BAX translocation to mitochondria. Mito-Q, Mito-TEMPOL, and Mito-Vit-E were shown to be more effective than untargeted antioxidants (vitamin E and ubiquinone10) at lower concentrations in protecting cells against peroxide-induced oxidative damage and death [146]. However, murphy et al. reported that a TPP^+^ concentration above 10 µM might have detrimental effects on mitochondrial damage control due to proton leakage and mitochondrial membrane depolarization [143].

#### 4.1.2. Rhodamine

Rhodamine derivatives have a high binding affinity for mitochondrial membranes so they can be used as agents that specifically target the mitochondria and disrupt the electron transport chain [154]. Rhodamine’s lipophilic and cationic characteristics, which enable it to pass through double mitochondrial membranes and stay inside the negatively charged mitochondrial matrix, are thought to constitute the mechanism causing its accumulation in mitochondria [155]. When TPP+ was replaced with rhodamine 19, it was found to be a potential drug carrier to mitochondria. This was accomplished by the chemical alteration of a TPP–drug conjugate so as to produce rhodamine 19–drug conjugates [147]. It inhibited stroke-induced brain swelling and averted neurological impairment, providing a potential example of a moderate uncoupler that is effective in the treatment of brain pathologies related to oxidative stress [156].

#### 4.1.3. Pyridinium Salts

Pyridinium salts are lipophilic delocalized cations that can be utilized as mitochondrial targeting groups. Large conjugated systems are created by modifying pyridinium salts with ethylenic bonds. The pyridine salts’ ability to withdraw electrons and their conjugated systems can induce or govern molecular luminescence, which is useful for the optical detection of mitochondria. However, pyridine salts’ lipophilicity hampers their ability to reach tissues in vivo.

A breakthrough in the use of pyridinium salts in vivo was made by Cheng’s team when they created a human serum albumin-facilitated pyridine salt complex that could easily access tumor tissue, had mitochondria-targeting ability, and caused tumor cell apoptosis [157]. The pyridine salt molecule’s cytotoxicity was a flaw in its mitochondrial detection, but it showed advantages in the treatment of tumors [157].

### 4.2. Mitochondria-Targeting Signal Peptides

The nuclear genome contains the genetic information for more than 1000 mitochondrial proteins. Cells usually utilize the mitochondrial protein import machinery to import nuclear-encoded mitochondrial proteins. These proteins typically have a 20–40-amino acid cleavable targeting region at their N-terminus. This sequence is generally positively charged, has amphiphilic helices, and is not conserved [158]. The movement of the imported proteins to the translocase of the inner membrane (TIM) complex at the IMM is carried out by the translocase of the outer membrane’s (TOM) importing complex, which is situated on the OMM. Following their passage through the IMM, mitochondrial peptidases break the targeting sequence, and mitochondrial chaperones fold the imported protein into a mature structure [159]. The majority of research efforts have focused on developing a mitochondrial targeting sequence for DNA or gene delivery into the mitochondria as a potential treatment to cure mitochondrial diseases [137,160]. This method is also utilized to investigate the precise involvement of mitochondria in the onset of some diseases, such as the modification of mitochondrial function and cell death caused by Aβ-peptide accumulation in mitochondria [161]. However, no prospective medicine including signal peptides for mitochondrial targeting has yet been evaluated, as this strategy has several practical limitations, i.e., effective targeting peptides are lengthy, large molecules are difficult to transport, the hydrophilicity and cellular permeability of these peptides are low, and the cost of chemical production is quite high. These limitations significantly restrict the application of mitochondria-targeted compounds (MTCs) based on mitochondrial targeting signal peptides. 

### 4.3. Cardiolipin Targeting (Penetrating Peptides)

Peptide-based delivery is an additional method utilized to facilitate mitochondrial targeting. The advantages of peptide-based delivery scaffolds are their simplicity in synthesis, adaptability, biocompatibility, and high absorption in both cells and in vivo [162]. The properties of lipophilicity and a positive charge were used to design small peptides with which to target mitochondria. Recent studies have shown that many cell-penetrating peptides can penetrate the mitochondrial membrane [163]. Szeto–Schiller (SS peptides) is an illustration of a small peptide-targeting moiety that targets mitochondrial cardiolipin with specificity in order to enhance mitochondrial plasticity and re-establish optimal bioenergetics. Based on their lipophilicity and charges, alternate aromatic residues and basic amino acids are being used to design such types of peptides to target mitochondria [164]. The accumulation of these peptides occurs specifically in the IMM, where they scavenge ROS, prevent the opening of the mitochondrial permeability transition pores, and, ultimately, prevent the release of cytochrome c. Various peptides (SS-01 to SS-31) were prepared by using these amino acids with slight modifications, from which (SS-01, SS-02, and SS-31) have shown antioxidant efficacy. This effect was due to the presence of aromatic amino acids, such as Dmt (dimethyl tyrosine) and Tyrosine, which subsequently scavenged ROS because unreactive tyrosyl or dityrosine radicals can react with superoxide radicals to form tyrosine hydroperoxide [138]. Contrary to TPP^+^, which causes toxicity at 10 µM, Szeto–Schiller peptides did not show toxicity even at 1 mM. Preclinical studies of SS-31(D-Arg-Dmt-Lys-Phe-NH2) suggest beneficial effects with respect to muscle aging, atherosclerosis, ischemia, osteoarthritis, diabetes, and glaucoma. To determine the efficacy and safety of these peptides, several clinical trials are undergoing for different diseases such as cardiovascular diseases, kidney diseases, cerebral ischemia, etc. [138,164].

Many other peptides such as cationic amphipathic α-helical D-(KLAKLAK)_2_ have also been synthesized in an attempt to improve the potencies of anticancer peptides [165,166]. In their study, Li et al. developed a peptide (P11LRR) consisting of arginine-modified polyproline amphiphilic molecules that formed a helical structure [167]. It was observed that the accumulation of P11LRR in mitochondria was driven by the mitochondrial transmembrane potential, as the elimination of mitochondrial potential leads to the inhibition of peptide localization. P11LRR-conjugated dimethyl tyrosine (a molecule that is supposed to be antioxidant) was able to reduce the chemically induced reactive oxygen species within the mitochondria, thereby serving as an excellent mitochondrial drug delivery vector [167]. Similar to this, various cell-penetrating peptides have been designed [168] despite having some drawbacks, such as fast elimination from the body, intracellular/vesicular entrapment, and non-specific internalization [169].

### 4.4. Nanoparticle (NPs)-Based Drug Delivery

#### 4.4.1. Dequalinium (DQA)

**Figure 4:**
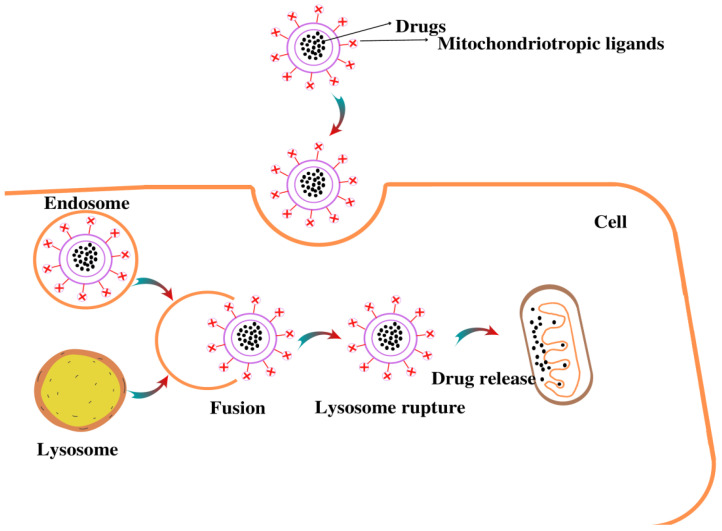
Illustration of mitochondria-targeting drug delivery by NPs.Drug loaded in liposomes undergoes endocytosis process, followed by formation of endolysosome. Once the endolysosome is ruptured, drug is released in the cytosol and targets the mitochondria.

Dequalinium, a single-chain amphiphilic compound with two delocalized cation centers that self-assemble into liposome-like cationic vesicles, has been shown to possess mitochondria-targeting characteristics [170]. It is capable of transporting DNA and drugs via nonspecific endocytic pathways to mitochondria, as shown in Figure 4. Various studies have suggested that DQA is capable of delivering DNA, and antisense RNA specifically, to the mitochondria [171,172]. Owing to this property, DQAsomes could also be utilized to encapsulate anti-cancer drugs such as curcumin and paclitaxel. These studies demonstrated that the free forms of curcumin and paclitaxel have less antioxidant and tumoricidal activity as compared to the encapsulated drug in DQAsomes [173,174]. DQAsomes are now considered to constitute a unique drug-delivery system due to the selective accumulation of the drug DQA in mitochondria and its anticarcinoma activity.

#### 4.4.2. Liposomes

Liposomes are globular structures formed from phospholipid-based vesicles that contain one or more lipid bilayers and cholesterol [175]. They are suitable for the incorporation of both hydrophobic and hydrophilic drugs due to their aqueous center and the lipid bilayer surrounding them. Liposomes are used to deliver drugs since they are biologically compatible, biodegradable, can self-assemble, nontoxic, can carry large drugs, and have several properties that can be changed to control their biological characteristics [176]. Drugs can be loaded into liposomes in a variety of ways, such as by encasing them in the aqueous region of the liposomes or lipophilic bilayers or by electrostatically adsorbing them to the liposome’s surface [177]. Various liposome systems have been developed for mitochondria-targeted delivery. In a study, chlorine e6 (Ce6, a photosensitizer) and IR 780 iodide (photothermal and near infra-red agent) were encapsulated in biocompatible liposomes and the attachment of TPP^+^ on the surfaces of liposomes greatly facilitated their mitochondria-targeted delivery and showed higher toxicity in HeLa cells in vitro, leading to the enhanced efficacy of photodynamic therapy [178]. Further research has been directed in this field and involved the use of the liposystem as a vehicle for the controlled release of a drug [179]. In another study, a stearyl residue was conjugated to TPP^+^ and this conjugated form was incorporated into a liposome. The ceramide-loaded stearyl-conjugated TPP liposomes (STPP) showed significantly decreased tumor volumes in BALB/c mice with non-specific toxicity [180]. For specific targeting, a novel polyethylene glycol-phosphatidylethanolamine (PEG-PE) conjugate was synthesized by attaching a TPP^+^ group to the distal end of the PEG block (TPP-PEG-PE). Further, this conjugate was encapsulated into a liposome and its toxicity, mitochondrial targeting capacity, and efficacy in delivering paclitaxel (PTX) to cancer cells were investigated in vitro and in vivo as compared to STPP conjugates. They found that the TPP-PEG-PE-modified liposomes were less cytotoxic and showed enhanced mitochondrial targeting capacity compared to the STPP-liposomes [181]. In another study, STPP-liposome hybrid cerasomes (CER), which are based on the Si–O–Si framework and liposomes, were developed to combat the instability and aggregation of the liposome system. Wang et al. synthesized this nano-hybrid cerasome modified with triphosphonium (TPP) and it showed excellent biocompatibility, good stability, and sustainable drug release behavior in mitochondria [182]. 

Yamada et al. created the MITO-Porter liposome to transport genome-targeting nucleic acids to the mitochondria. It is a liposome-based transporter that effectively transports molecules to the cytoplasm [183,184], as well as to mitochondria through membrane fusion [185]. High-density octa-arginine (R8) was used by Yamada et al. to coat the MITO-Porter surface, which led to macropinocytosis rather than clathrin-mediated endocytosis and enabled particles to reach the cell without being harmed. Later, Yasuzaki et al. employed MITO-Porter to encapsulate propidium iodide, a fluorescent dye used for staining nucleic acids that could enable the visualization of mtDNA. Further investigation into this strategy is being conducted for photodynamic cancer therapy [186] and mitochondrial gene therapy [187,188].

#### 4.4.3. Polymeric Nanoparticles

Polymeric NPs could be used to target mitochondria for drug delivery since they are biodegradable and biocompatible. Additionally, as they are simple to make, surface alterations can be performed easily and can be customized to drug-release characteristics. Various polymers such as poly (lactic-co-glycolic acid) (PLGA), poly (glycolic acid) (PGA), poly (lactic acid) (PLA), and polycaprolactone (PCL) can be formed into nanoparticles through emulsification-solvent evaporation or nanoprecipitation [189,190]. These NPs can encapsulate both hydrophilic and hydrophobic drugs with minor modification of FDA-approved hydrophilic blocks such as polyethylene glycol (PEG). These PEG molecules augment the residence time in vivo and are generally used to conjugate the targeting moieties [2]. Through various chemical reactions, numerous nanocarriers have been made for drug delivery. To assess the use of these nano-carriers, different drugs have been employed as their payloads, including 2, 4-dinitrophenol (an anti-obesity treatment), curcumin (AD), and α-tocopheryl succinate (a cancer medication) [191]. In comparison to non-targeted constructs or treatments in their free form, the drug therapeutic index for cancer, AD, and obesity is noticeably improved by targeted nanocarriers such as PLGA-b-PEG-TPP NP [191].

#### 4.4.4. Inorganic Nanoparticles

Inorganic nanoparticles have a smaller and more homogeneous particle size than organic NPs. In contrast to liposomes, dendrimers, and micelles, metallic nanoparticles (Metal NPs) such as silver and gold have features including surface plasmon resonance (SPR), enhanced Rayleigh scattering, and abilities beneficial for imaging biological systems [192]. These metallic nanoparticles can be easily conjugated with peptides, antibodies, DNA, and RNA to target certain cells, and with biocompatible polymers (polyethylene glycol) to extend their circulation in vivo for medication and gene delivery applications. Therefore, metal NPs have been employed as core components because of their bio-inertness, simplicity in synthesis, and characterization.

**Figure 5:**
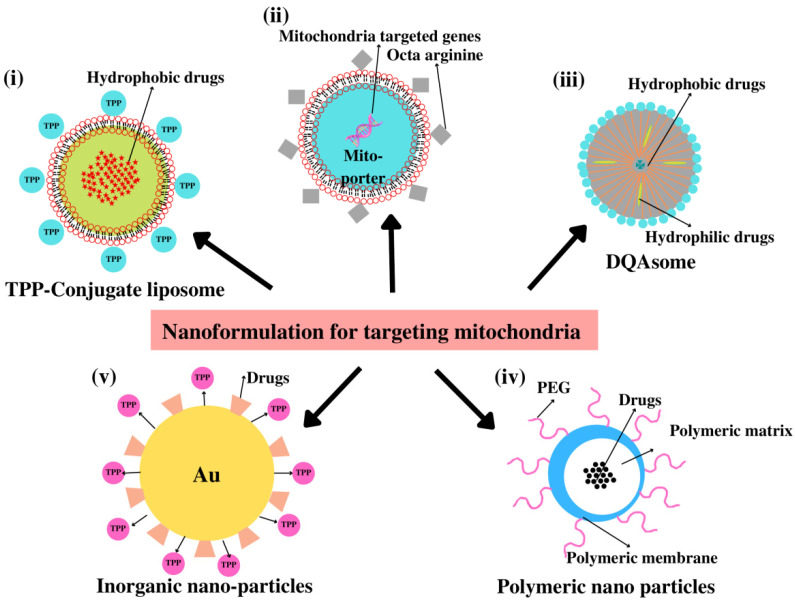
Different nanoformulations for mitochondria-targeted drug delivery. (**i**) Loading of hydrophobic drugs into the TPP^+^-conjugated liposome. (**ii**) Mito-porter containing mitochondria-targeting gene with surface modified with octa-arginine. (**iii**) Loading of hydrophobic and hydrophilic drugs in DQAsome. (**iv**) Drug loaded in polymeric NP modified with PEG in mitochondrial delivery. (**v**) Gold NP loaded with drugs in conjugation with mitochondriotropic ligand TPP^+^.

The use of silver nanoparticles (AgNPs) as nanocarriers for the treatment of cancer has shown promising results [193]. They possess special antiviral, antibacterial, and antimicrobial activities. Additionally, AgNPs, with or without conjugates, have been recognized for their anticancer activities, which makes them effective drug carriers for the treatment of cancer [23]. AuNPs have been used to provide targeted medication delivery by conjugating with various mitochondrial moieties. We have summarized the different mitochondria-targeting nanoformulations in Table 1. In the human cancer cell lines, Caco-2, HeLa, and MCF-7, Oladimeji et al. investigated the mitochondria-targeted delivery of betulinic acid (BA) via mitochondriotropic TPP^+^-functionalized epigallocatechin gallate (EGCG)-capped gold NPs (AuNPs). The IC50 values of these nanocomplexes were 3.12–13.2 µM in vitro, compared to 9.74–36.31 µM for free BA, and they likewise demonstrated a considerable reduction in cancer cell proliferation [194]. The majority of traditional chemotherapeutics have harmful side effects that restrict the maximum tolerable dose and compromise their therapeutic efficiency by indiscriminately killing both healthy and malignant cells. To solve this issue, Sun et al. created the DNA-guided missile-integrated nanospacecraft (GM-NSC), a nanocomposite formed of gold nanoparticles (AuNPs) and a high-density multilayer DNA crown that is constructed from highly organized DNA tetrahedral units (DNA Tetra). Each DNA tetrahedral unit consists of three parts: an explosive bolt (E-bolt), a triphenylphosphonium (TPP) unit that targets mitochondria, and an aptamer that targets cancer cells [195]. Good biocompatibility, high cargo-loading capacity, adequate in vivo biodistribution, and therapeutic efficiency without side effects were demonstrated by GM-NSC, making it a viable alternative drug delivery system for targeted cancer treatment [195]. Figure 5 depicts the various nanoformulations currently in use for mitochondria-targeted drug delivery.

## 5. Conclusions

Mitochondria are among the most important cellular organelles, which, in addition to energy production, are involved in calcium signaling, cell growth and differentiation, cell cycle regulation, and apoptosis. Mitochondrial dysfunction is a potential target for diagnostic and therapeutic interventions for many diseases owing to its role concerning higher cell death, decreased ATP synthesis, and increased mtROS production in the pathogenesis of many diseases. The mitochondrial membranes and plasma membranes restrict therapeutic molecules’ ability to reach mitochondria. Although there is a bioactive additive with MitoQ and eye drops with SkQ1, there is still a scarcity of pharmacological formulations in the market that efficiently target mitochondria. The reason for which is the lack of an effective delivery system that can direct therapeutic molecules for selective accumulation inside mitochondria. The ability of NPs to localize within mitochondria and target particular cells facilitates treatments associated with mitochondrial dysfunction disorders. Nano-formulation approaches, such as those incorporating liposomes, inorganic NPs, and polymeric NPs conjugated with mitochondriotropic ligands, have been shown to carry a variety of payloads to mitochondria in in vitro models, although several clinical and pre-clinical studies still need to be conducted to understand the safety of these drug-delivery systems. The field of mitochondria-targeted nanomedicine is gaining considerable interest and efforts are also being made for the preparation of potent nano-formulations; however, a better understanding of the subject is still needed in order to use them as potential therapeutics for a wide range of diseases.

## Figures and Tables

**Table 1:** Summary of mitochondria-targeting nanoformulations.

Vector	Mitochondriotropic Moiety	Drug/Cargo	Model	Effect	Ref.
Liposomes	TPP^+^	Paclitaxel, Doxorubicin	Hela and 4T1 cancer cell line	Shows enchanced cytotoxicity to cancer cells as compared to free drug, antitumor activity, and high cell uptake efficiency	[181,196]
Liposomes	MITO-Porter	Coenzyme Q10, Doxorubicin	Mouse liver ischemia/reperfusion injury (I/R injury) model, OS-RC-2 cells	Decreases the level of alanine amino-transferase, antitumor activity	[197,198]
Liposomes	DQA	Resveratrol	Human lung adenocarcinoma A549 cells and resistant A549/cDDP cells	Induced apoptosis in both cell lines via mitochondria	[199]
Liposomes	DQA	Topotecan	Breast cancer MCF-7 and resistant MCF-7/adr cells	Shows enhanced accumulation in mitochondria and anti-cancer effect	[200]
Liposomes	Rh123	Paclitaxel	Hela and B16-F10 cancer cell line	Induced apoptosis and high toxicity to cancer cells	[201]
Liposomes	STPP^+^	Ceramide	4T1 mammalian carcinoma cells and animal model	Enhanced specific drug delivery, show anti-tumor effect	[180]
Cerasomes	TPP^+^	Doxorubicin	Hela cells	Sustainable drug release, high biocompatibility, show antitumor effect	[182]
AuNP (Polydopamine)	-	Paclitaxel	Cancer cell line	Downregulates anti-apoptotic gene, enhanced anti-cancer efficacy	[202]
AuNP (Hyaluronic acid)	-	Camptothecin	Cancer cell line	Upregulates proapoptotic genes, sensitizes drug-resistant cancer cells, enhanced anti-cancer efficacy	[203]
PLGA-PEG NPs	TPP^+^	Curcumin, 2,4-dinitrophenol, lonidamine, α-tocopheryl succinate	HeLa cells	Enhanced specific drug delivery, enhanced cytotoxicity to cancer cells	[191,204]
Mesoporous silica NPs	TPP^+^	α-tocopheryl succinate	HeLa and HepG2 cancerous cell line	Enhanced cytotoxicity, disrupt mitochondrial membrane potential	[205]
AgNPs (Polydopamine)	RGDARF peptide (Tumor targeting peptide)	Paclitaxel	Cancer cell line	Strong apoptotic-inducing potency, activation of pro-apoptotic factor P53 and caspase 3	[193,206]

## Data Availability

The information that supports the findings of this study is available in this article.

## Abbreviations

IMM	Inner mitochondrial membrane
OMM	Outer mitochondrial membrane
IMS	Intermembrane space
TPP	Triphenylphosphonium
Dmt	Dimethyltyrosine
DQA	Dequalinium
MitoE	TPP-vitamin E
Mito-Q	TPP-ubiquinone
STPP	Stearyl conjugated TPP liposomes
PEG	Polyethylene glycol
PTX	Paclitaxel
PLGA	Poly (lactic-co-glycolic acid)
BCL-2	B-cell lymphoma
ALS	Amyotrophic lateral sclerosis
PD	Parkinson’s disease
AD	Alzheimer’s disease
MS	Multiple sclerosis
